# STEAM-DWI as a robust alternative to EPI-DWI: Evaluation in pediatric brain MRI

**DOI:** 10.1371/journal.pone.0268523

**Published:** 2022-05-18

**Authors:** Daniel Gräfe, Anne Päts, Andreas Merkenschlager, Christian Roth, Franz Wolfgang Hirsch, Jens Frahm, Dirk Voit

**Affiliations:** 1 Department of Pediatric Radiology, Leipzig University, Leipzig, Germany; 2 Department of Pediatrics, Leipzig University, Leipzig, Germany; 3 Biomedical NMR, Max Planck Institute for Multidisciplinary Sciences, Göttingen, Germany; Linköping University, SWEDEN

## Abstract

**Purpose:**

Diffusion-weighted imaging (DWI) is an essential element of almost every brain MRI examination. The most widely applied DWI technique, a single-shot echo-planar imaging DWI (EPI-DWI) sequence, suffers from a high sensitivity to magnetic field inhomogeneities. As an alternative, a single-shot stimulated echo acquisition mode diffusion-weighted MRI (STEAM-DWI) has recently been re-introduced after it became significantly faster. The aim of the study was to investigate the applicability of STEAM-DWI as a substitute to EPI-DWI in a daily routine of pediatric radiology.

**Methods:**

Retrospectively, brain MRI examinations of 208 children with both EPI-DWI and STEAM-DWI were assessed. Visual resolution and diagnostic confidence were evaluated, the extent of susceptibility artifacts was quantified, and contrast-to-noise ratio was calculated in case of diffusion restriction. Furthermore, the correlation of apparent diffusion coefficient values between STEAM-DWI and EPI-DWI was tested.

**Results:**

STEAM-DWI was inferior to EPI-DWI in visual resolution but with higher diagnostic confidence and lower artifact size. The apparent diffusion coefficient values of both sequences demonstrated excellent correlation. The contrast-to-noise ratio of STEAM-DWI was only half of that of EPI-DWI (58% resp. 112%).

**Conclusion:**

STEAM-DWI is a robust alternative to EPI-DWI when increased susceptibility artifacts are to be expected. Drawbacks are a lower contrast-to-noise ratio and poorer visual resolution.

## Introduction

Diffusion-weighted imaging (DWI) is an essential element of almost every brain MRI examination. It yields additional information, especially in cases of infection, ischemia, neoplasia, epilepsy, demyelinating diseases and encephalopathies [1]. The technique of choice for several decades has been a single-shot echo-planar imaging sequence (EPI-DWI). Despite continuous technical advances, its strongest intrinsic limitation remains its susceptibility to magnetic field inhomogeneities, e.g. in the vicinity of metal such as surgical or dental implants and tissue to air junctions, leading to both geometric image distortions and altered intensities with signal pile-ups or dropouts [2]. In particular, the susceptibility to metal is a relevant problem, especially for the pediatric population, since along with dental prosthetic material, valves from ventriculoperitoneal shunting, pressure-sensitive probes after neurosurgical interventions and the increasing number of MR conditional cochlear implants often limit the reliability of EPI-DWI.

A solution to this dilemma has been offered by combining a single-shot stimulated echo acquisition mode (STEAM) MRI sequence [3] with a leading spin-echo diffusion module (STEAM-DWI) [4]. However, the sequence has not been widely used so far due to its intrinsically low signal-to-noise ratio (SNR). Only recently, owing to modern acquisition and reconstruction techniques borrowed from real-time MRI, has it become possible to perform STEAM-DWI with a favorable SNR in a decent acquisition time [5, 6].

There is little experience regarding its clinical applicability, particularly in children. Furthermore, there is no systematic comparison of image quality and apparent diffusion coefficient (ADC) values between STEAM-DWI and EPI-DWI. The aim of the study was to compare the clinical value of both sequences in a pediatric population.

## Methods

### Patients

The retrospective study included patients between 0 and 18 years of age who had undergone brain MRI with EPI-DWI and STEAM-DWI for clinical indication between February and July 2020. The study was approved by the ethics committee of the University Hospital Leipzig (209/20-ek). Written informed was waived due to the retrospective nature of the study and due to the anonymization of data. All data are available from paedrad@uniklinik-leipzig.de for researchers upon reasonable request.

### MRI acquisition

MRI was performed at 3T on a standard clinical scanner (Prisma fit, Siemens, Erlangen, Germany) equipped with a dedicated computer for online reconstruction of diffusion-weighted images and ADC maps as described [6]. In brief, the technique combines a diffusion-weighted (DW) spin-echo module and a STEAM MRI readout with undersampled radial trajectories and covers a volume by a series of gapless cross-sectional slices. In a first step, optimal coil sensitivities for all slices are obtained from a series of non-DW acquisitions by nonlinear inverse reconstruction with regularization to the image and coil sensitivities of a directly neighboring slice. In a second step, these coil sensitivities are used to compute all series of non-DW and DW images by linear inverse reconstruction with spatial regularization to a neighboring image. The reconstruction of the whole stack finished only a few seconds after the end of image acquisition. A pediatric 16-channel head-neck coil was used for infants in the first year of life, and a 64-channel head coil was selected for older children and adolescents (S1 Fig). The parameters for EPI-DWI and SEAM-DWI are presented in Table 1.

**Table 1:** Parameters for EPI-DWI and STEAM-DWI.

	EPI-DWI	STEAM-DWI
**Field-of-view (mm)**	220 (infants 180)	224 (infants 160)
**Voxel-size (mm)**	1.2 x 1.2 x 3.0 (interpolation 0.6 x 0.6 x 3.0)	1.0 x 1.0 x 3.0
**TR / TE (ms)**	6600 / 81	10.000 / 36
**Bandwidth (Hz/px)**	1462	230
**Diffusion directions**	4	6
**b-values (averages)**	b0 / b1000 (2 / 2)	b0 / b1000 (3 / 2)
**Parallel Imaging**	PAT 2 (GRAPPA)	n/a
**Duration for 50 sections covering a 15 cm volume (min)**	1:26	2:25

### Image analysis

EPI-DWI and STEAM-DWI were compared according to five criteria: 1) resolution 2) diagnostic confidence 3) extent of artifacts 4) contrast-to-noise ratio (CNR) and 5) absolute ADC values. Evaluation was performed by two experienced readers (D.G. with 12 years and A.P. with 5 years of experience in pediatric imaging). The evaluation of the sequences was performed with a standard DICOM viewer (Intellispace Portal 10.1, Philips, Best, The Netherlands).

1. Resolution: In every patient, visibility of the mesencephalic aqueduct (usually around 1.5 mm in diameter) was ranked on a Likert scale (1 = not visible; 2 = blurry; 3 = sharp visualization) (S2 Fig).
2. Diagnostic confidence: In every patient, both DWI sequences were ranked on a Likert scale (1 = at least one ambiguous signal; 2 = at least ambiguous signal to only the unexperienced reader; 3 = full diagnostic confidence) (S3 Fig).
3. Dimensions of artifacts: In patients with ventriculoperitoneal shunt valves, a freehand region of interest was placed on brain tissue obscured by the artifact on single slice position with the greatest artefact dimensions. These artifacts manifested as morphological distortions as well as a regionally increased cortex signal in the isoDW image (S4 Fig).
4. CNR in both sequences was determined in a subpopulation of children with areas of diffusion restriction. Diffusion restriction was defined as increased signal in the isoDWI image in combination with a corresponding signal decrease in the ADC map. Freehand regions of interest (ROIs) were drawn as large as possible but omitting the outermost voxels of the lesion. A minimal area of 10 mm^2^ was required. The magnitude of gain in intensity of a lesion compared to healthy tissue can be expressed as percentage CNR [7]. Percentage CNR was calculated for STEAM-DWI and EPI-DWI by creating a ROI on the respective isoDWI image as ratio of intensity of the diffusion-restricted lesion to a matched normal contralateral area as control. Thus, percentage CNR was calculated using the formula: (lesion—control)/control [8] (S5 Fig).
5. ADC values were determined in a subpopulation of children without major susceptibility artifacts as defined beyond common artifacts at the bone and air interfaces, predominantly frontopolar and temporopolar and infratentorial. The actual level of diffusion can be quantified by the ADC map. Because the validity of such a correlation mainly benefits from the broad scatter of the samples over the whole range of diffusivity and since it was not intended to assess specific brain regions, the ROIs were not drawn in a structured manner into predefined regions but were set specifically by the observer to ensure a corresponding acceptable scatter of the ADC values between 600 and 1600 (10^−6^ mm^2^ /s). For comparison of ADC values between STEAM-DWI and EPI-DWI, multiple ROIs were drawn in regions with homogeneous signal of ADC map of the STEAM DWI. This ROI was transferred to the anatomically identical region of the other sequence by applying the conventional copy and paste function of the ROI of the DICOM Viewer. The correct matching of the region was verified using a T2 TSE sequence and for homogeneity on the ADC map. To correlate the ADC values of the STEAM-DWI with the ADC values of the EPI-DWI, freehand ROIs were drawn at the same position in both sequences. The following requirements were met in the selection of the ROI: homogeneous ADC signal, minimal ROI area of 10 mm^2^, supratentorial brain regions, at least 2 cm spacing distance to the skull. Areas in the vicinity to amorphological distortion or other susceptibility artifact as well as cerebrospinal fluid were omitted (S6 Fig).

The degree of correlation of the ADC values was determined using Pearson’s correlation coefficient. Correlation between the two readers was evaluated with Cohen’s kappa for ordinal data and intraclass correlation coefficient for interval data. Differences between groups were compared using the Wilcoxon rank sum test when normal distribution in the Shapiro-Wilk test was unlikely. Statistical analysis was performed using RStudio (RStudio v1.2.5033, RStudio Inc., Boston, MA).

## Results

### Patients

In total, brain MRI was reviewed in 208 patients (median age 10.8 years (interquartile-range 4.7–14.9 years); 107 male, 17 patients below 1 year of age) who received both EPI-DWI and STEAM-DWI. Of these, 33/208 (15.8%) revealed major susceptibility artifacts in EPI-DWI. This was caused by braces (n = 5), valves from a ventriculoperitoneal shunting (n = 10), surgical material after craniotomy (n = 16), hemosiderin (n = 1), and pneumencephalus (n = 1). Restricted diffusion was detected in 18/208 patients (8.7%). Causes for restricted diffusion in these patients were perioperative damage (n = 10), trauma (n = 4), glioma, multiple sclerosis, adrenoleukodystrophy and perinatal hypoxia.

### Resolution, diagnostic confidence, and artifact dimensions

**Fig 1:**
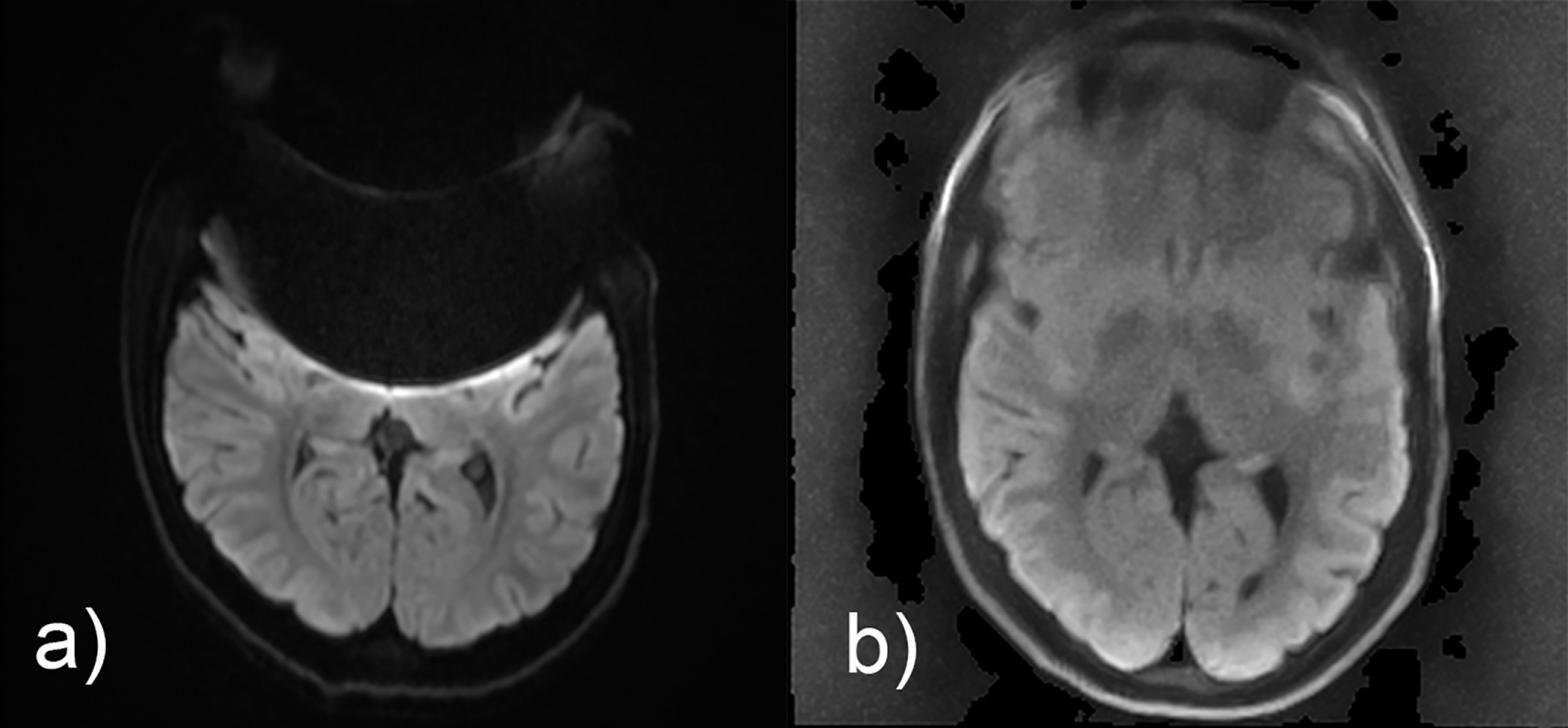
Comparison of isoDW images obtained by (a) EPI-DWI and (b) STEAM-DWI in a 15-year-old adolescent with epilepsy without pathological findings. A brace leads to severe frontal susceptibility artifacts, which are much less pronounced in STEAM-DWI.

**Fig 2:**
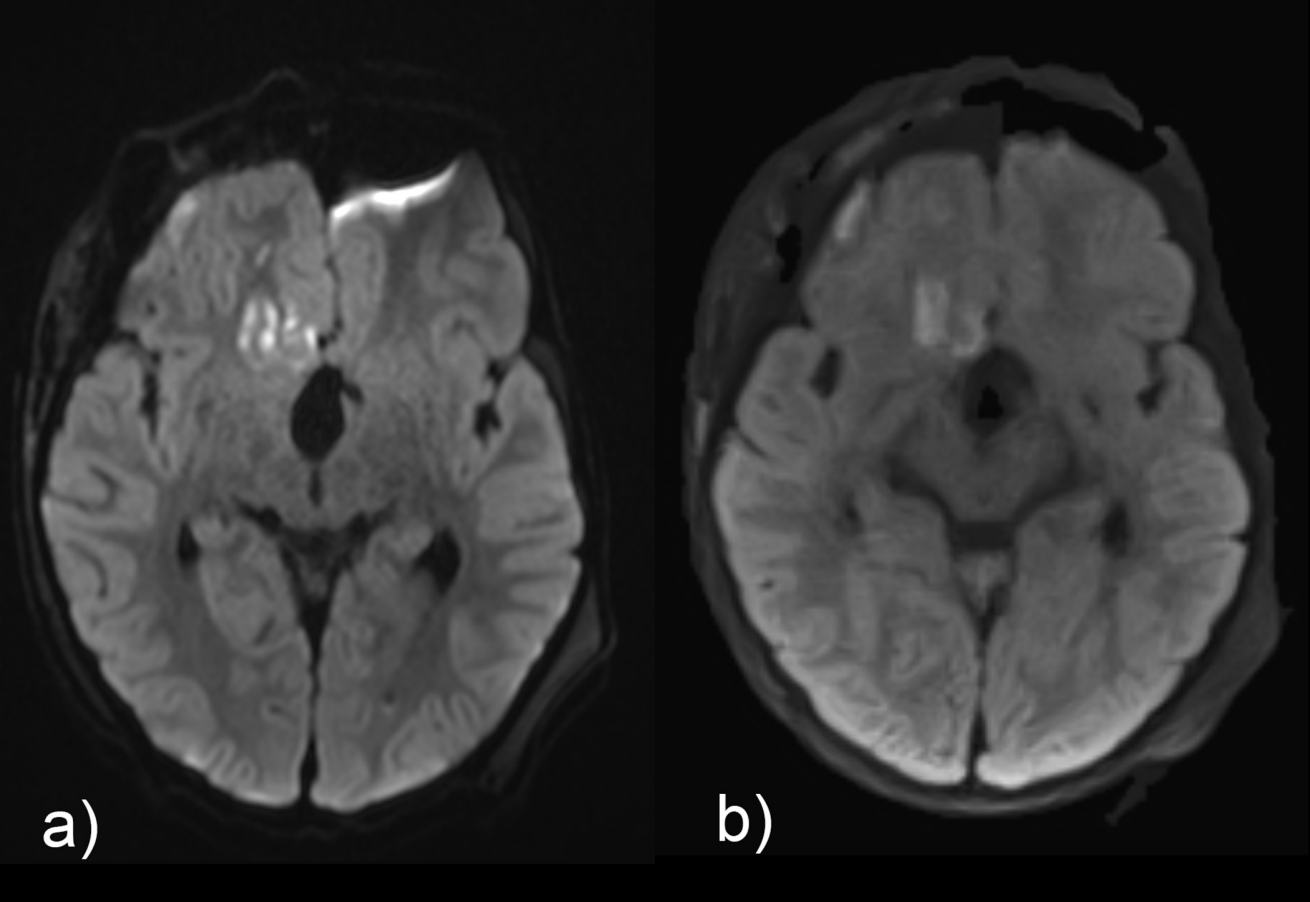
Comparison of isoDW images obtained by (a) EPI-DWI and (b) STEAM-DWI in a 4-year-old boy with status post resection of craniopharyngeoma. In contrast to EPI-DWI, the left-frontal pneumencephalus does not cause artifacts with STEAM-DWI. Please note the hyperintense changes in isoDWI which showed restricted diffusion in ADC, in the right hemisphere in both the fontal cortex and frontal white matter.

**Fig 3:**
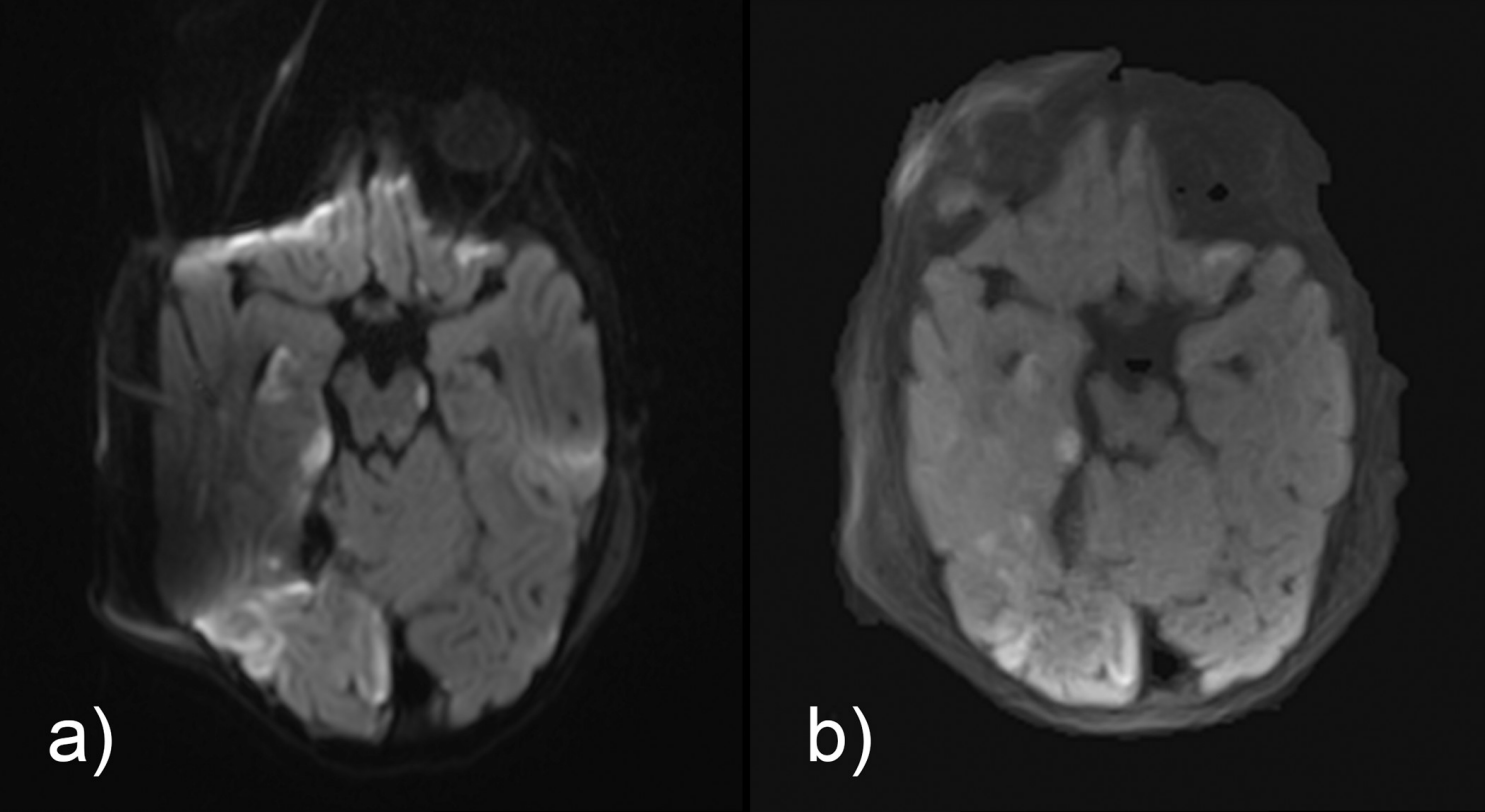
Comparison of isoDW images obtained by (a) EPI-DWI and (b) STEAM-DWI in a 14-month-old girl with posttraumatic epidural hemorrhage and partial infarction in the area of the right posterior cerebral artery (postoperative situs). Susceptibility effects due to the surgical foreign material on the calvaria and an intracranial pressure probe (in adjacent layer) result in moderate distortion and hyperintensity artifacts, which are not noticeable in STEAM-DWI. Please note that the hyperintense alterations in the EPI-DWI right frontal are exclusively due to artifacts. Depiction of the small hyperintense spot in the left crus cerebri in STEAM-DWI is inferior compared to EPI-DWI.

**Fig 4:**
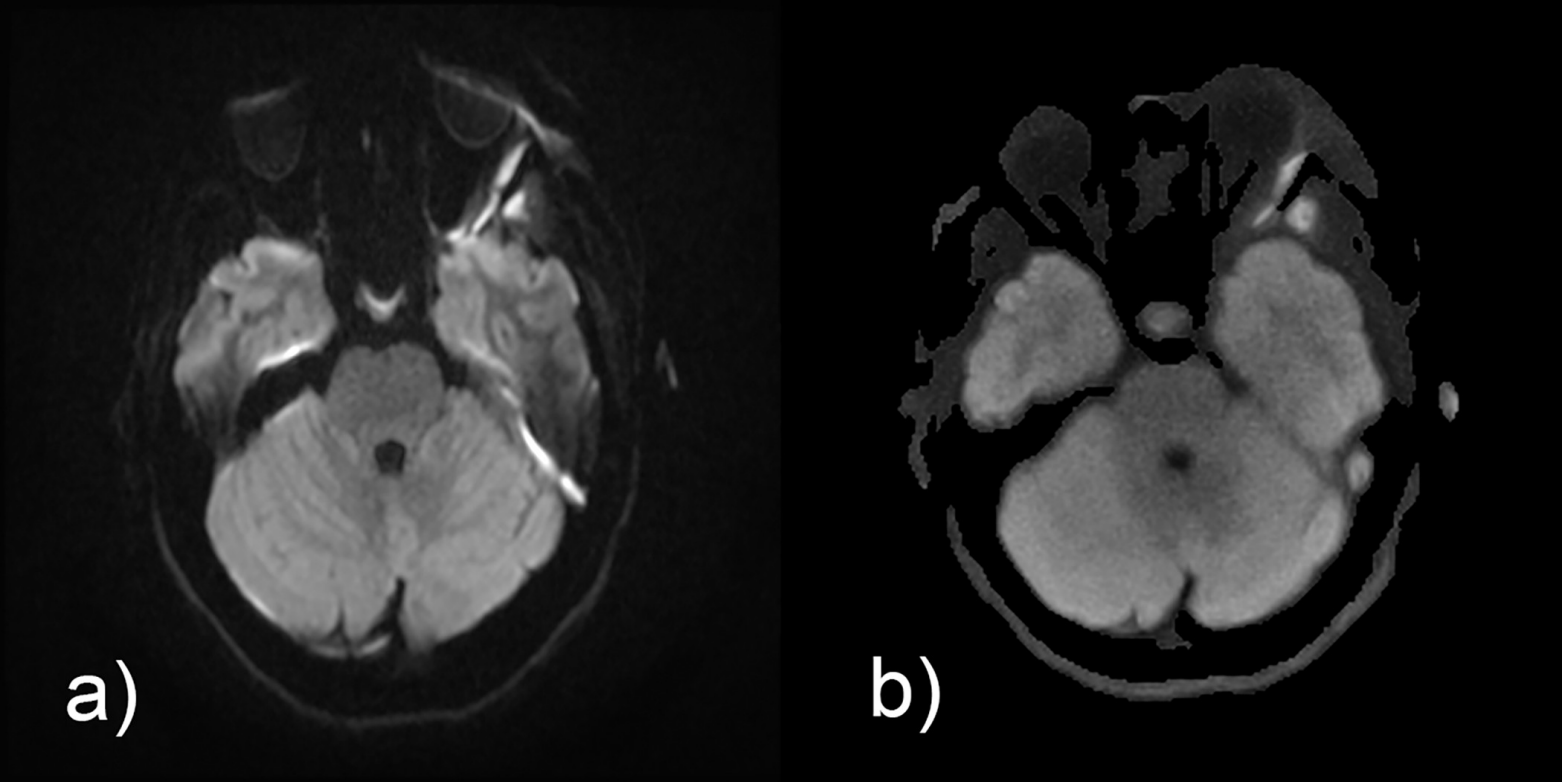
Comparison of isoDW images obtained by (a) EPI-DWI and (b) STEAM-DWI in a 13-year-old girl with left-sided postseptal inflammation and abscess lateral to the rectus lateralis muscle into the temporalis muscle. The diffusion restriction can be seen in both EPI-DWI and STEAM-DWI, but the morphological distortions and artificially increased signal at the anterior and posterior border of the conus make it difficult to accurately assess the extent of the abscess in EPI-DWI.

STEAM-DWI showed lower visual resolution (median score of 1 and 3, respectively, p < .001, interreader reliability κ>0.81) but higher diagnostic confidence (median score of 3 and 2, respectively, p < .001, κ>.82) compared to EPI-DWI (Tables 2 and 3). For shunt valves, the area missed by susceptibility artifacts was in median 35.5% (IQR 2.5% - 67.7%, p < .001) smaller with the STEAM DWI than with the EPI-DWI (ICC 0.99). Pronounced field distortions, as caused by braces, resulted in considerably smaller areas of signal void and no morphological distortions at neighboring regions (Fig 1), though no statistical analysis has been performed due to the small number of subjects within each subgroup. Mild artifacts, e.g., due to hemosiderin or a pneumencephalus, were prevented completely in STEAM-DWI (Figs 2–4).

**Table 2:** Visual judgment of resolution for EPI-DWI and STEAM-DWI on a Likert scale (range 1 to 3) in 208 patients.

	1	2	3
**STEAM-DWI**	137 (65.9%)	70 (33.7%)	1 (0.5%)
**EPI-DWI**	0 (0%)	1 (0.5%)	207 (99.5%)

**Table 3:** Diagnostic confidence for EPI-DWI and STEAM-DWI on a Likert scale (range 1 to 3) in 208 patients.

	1	2	3
**STEAM-DWI**	2 (1.0%)	0 (0%)	206 (99.0%)
**EPI-DWI**	13 (6.3%)	195 (93.8%)	0 (0%)

### CNR and ADC values

**Fig 5:**
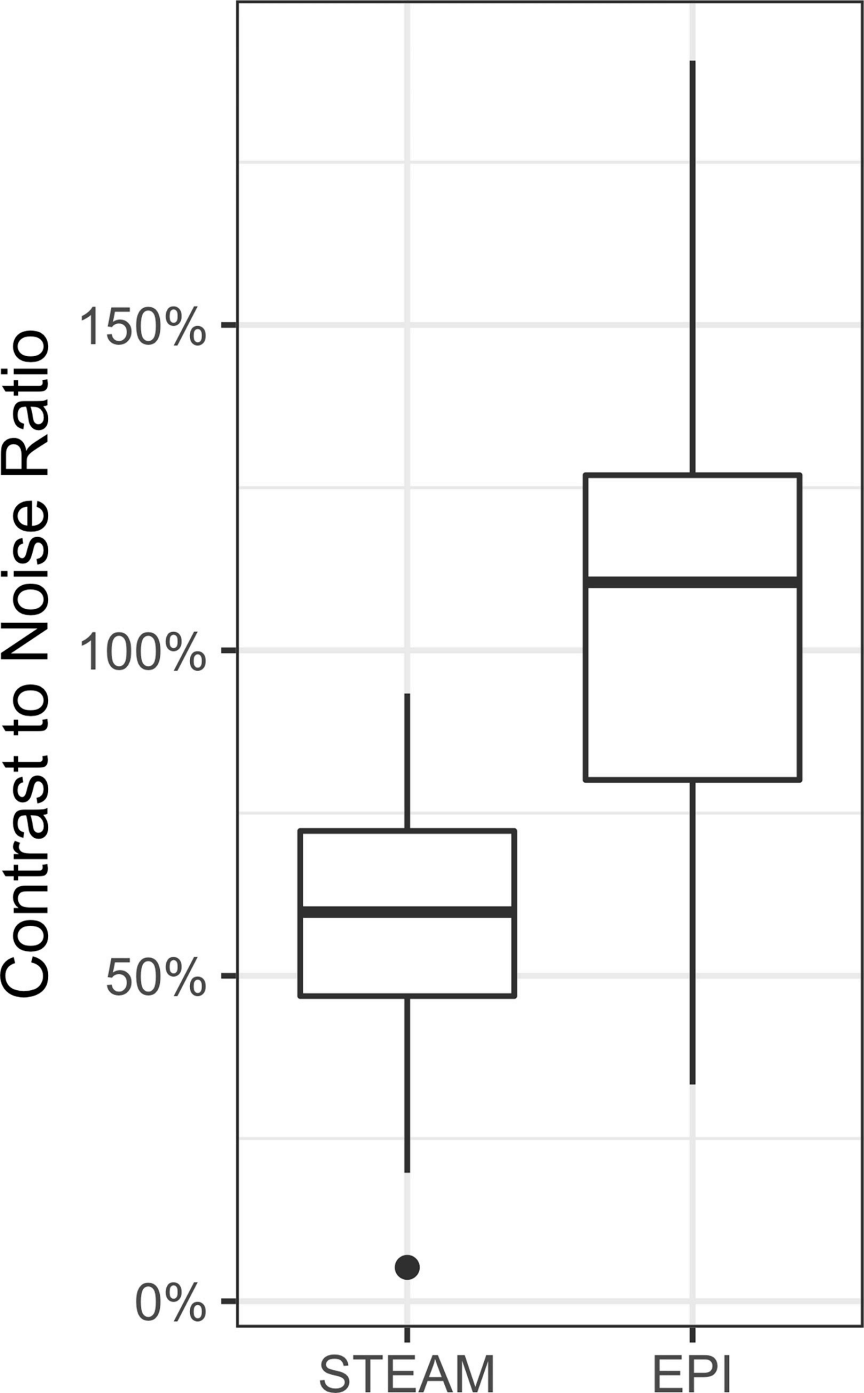
The contrast-to-noise ratio in isoDW images of STEAM-DWI is only about half that of EPI-DWI (mean CNR 58% vs. 112%).

**Fig 6:**
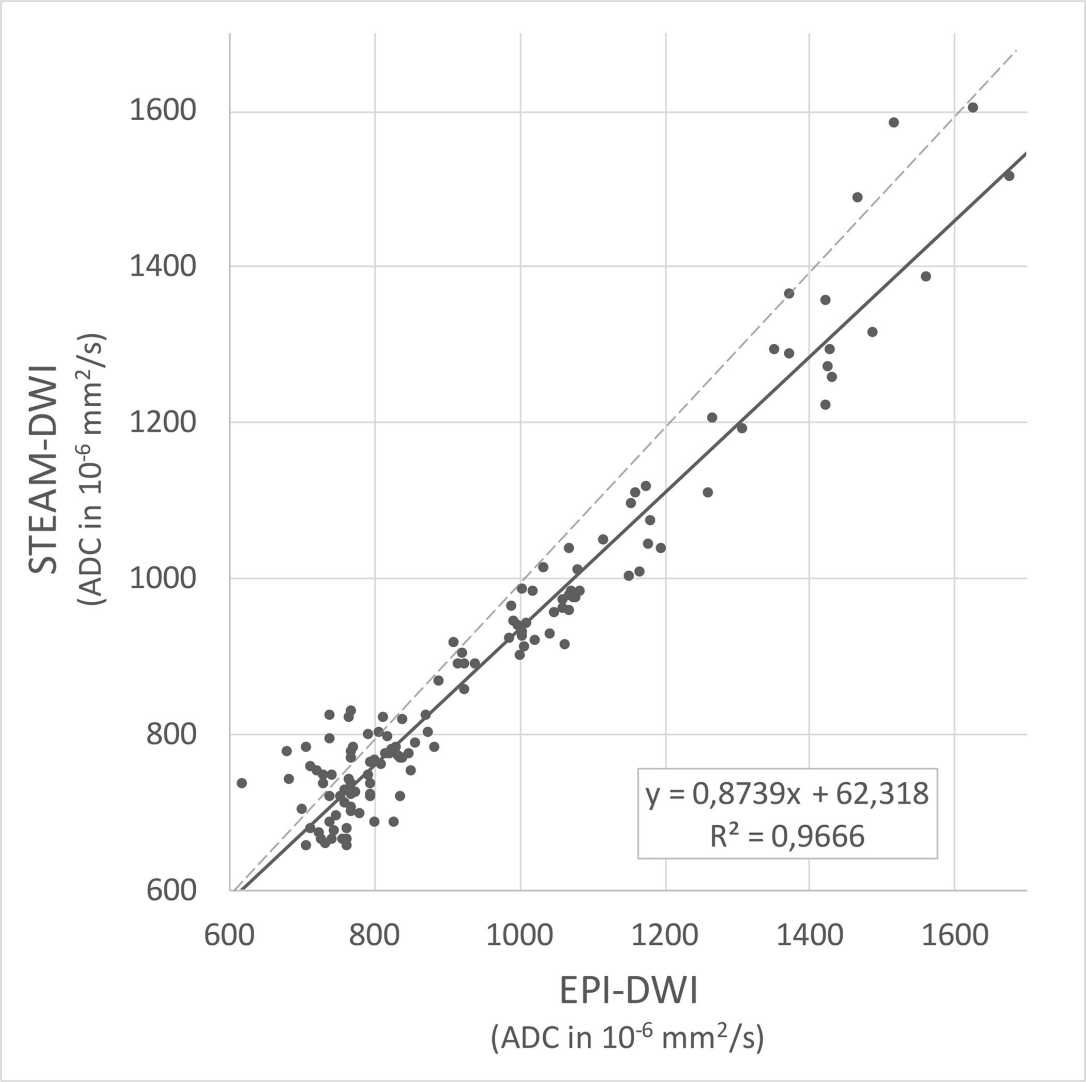
Corresponding ADC values as randomly obtained by EPI-DWI and STEAM-DWI. On average (solid black line), STEAM-DWI values are 13% lower than those obtained by EPI-DWI (medians as dashed black line).

A total of 37 ROIs from 18 patients were placed in different diffusion-restricted lesions, and the CNR was calculated for both DWI sequences. The mean CNR of EPI-DWI was 112% (standard deviation 33%), which exceeded that of STEAM-DWI with 58% (standard deviation 20%) (p < .001) (Fig 5). For comparison of the absolute ADC values, 141 samples were randomly taken in 184 patients without major susceptibility artifacts. The ROIs were obtained in homogeneous parenchyma regions and then transferred with respect to size and position to the second diffusion sequence. There was excellent, essentially linear correlation (R^2^ = 0.97) of the ADC values between the two sequences, with the ADC values of STEAM-DWI being 13% lower than those of EPI-DWI (Fig 6 and S7 Fig).

## Discussion

There are several approaches to circumvent geometric image distortions and artificial signal alterations resulting from magnetic field inhomogeneities, which are particularly pronounced in EPI-DWI [2]: First, effective echo-spacing may be decreased by parallel imaging. Second, the phase-encoding direction can be switched to alter the propagation of erroneous signal. Furthermore, single-shot gradient-echo acquisitions can be replaced by multi-shot acquisitions, resulting in interleaved EPI sequences (e.g., multiplexed sensitivity encoding or MUSE [9]), readout segmented-EPI (RESOLVE [10]), short-axis propeller-EPI as well as spiral-readout sequences. These multi-shot sequences can mitigate the shortcomings of single-shot EPI-DWI to a certain degree but generally do not eliminate them entirely. Another approach to robust diffusion sequences relies on Fast Spin Echo (FSE) DWI sequences (e.g., DWI-PROPELLER [11]), which employ a train of refocusing radiofrequency pulse with high flip angles to compensate for signal alterations due to magnetic field inhomogeneities. Thus, the trade-off in FSE DWI sequences has been their elevated specific absorption rate. A recent and comprehensive review of common diffusion sequences and their limitations was published by Holdsworth et al. [2].

In STEAM-DWI, the general encoding pattern is the same as in most other DWI sequences, namely by means of a diffusion module which consists of a spin-echo interval with self-compensated magnetic field gradients. However, instead of an EPI or FSE readout element, single-shot STEAM-DWI reads out by a series of stimulated echoes with low flip-angle RF pulses which in the present study allow for the acquisition of a highly undersampled radial image. In conjunction with an iterative inverse reconstruction with spatial regularization [6], this data provides diffusion-weighted high-speed images which are only slightly affected in the presence of strong magnetic field distortions. While earlier implementations based on Fourier imaging [4] failed to be adopted in routine diagnostics due to their low SNR, a more recent applications of a preceding version of the current technique revealed promising clinical results [5].

The benefits of a DWI sequence that is barely affected by susceptibility differences are obvious: (i) As there are no morphological distortions, diffusion-restricted regions can be better correlated with the mainstay of brain MRI, i.e. T1 or T2 weighted images. (ii) There are no signal pile-ups and drop-outs at interfaces with different magnetic susceptibilities, which can lead to ambiguities in the interpretation of DWI, especially for inexperienced radiologists. (iii) The area of signal loss due to metallic implants is much smaller (in our study, it is 35% as an example in shunt valves), thus enlarging the assessable brain area. For the abovementioned reasons, studies on the clinical applicability of STEAM-DWI are scarce and only exist in the adult age group. In 2016, Khalil et al. were able to demonstrate a reduced artifact level with the same sensitivity in the detection of infratentorial ischemia with STEAM-DWI compared to EPI-DWI [7].

Our study indicates that the measures of diffusivity in STEAM-DWI correlate linearly with those of EPI-DWI. The systematic deviation of the absolute ADC values by about 13% between the two techniques is not surprising: Even two EPI-DWI sequences with identical parameters exhibit a standard deviation of the ADC values of up to 9% for different MRI manufacturers, models and sites [12]. Therefore, in general, ADC values obtained from the literature can only be applied with caution for the neuroradiological differentiation of various disease entities, such as brain tumors [13]. Moreover, in the present work, STEAM-DWI employed six diffusion gradient directions to better deal with diffusion anisotropy, whereas EPI-DWI used only four. It is not quite clear how this slightly more accurate sampling of anisotropic diffusion properties alters mean ADC values. In addition, further influences may be due to the use of different spin-echo times for diffusion weighting (TE 81 ms for EPI-DWI vs TE 36 ms for STEAM-DWI) which, even for identical b values, may lead to different gradient amplitudes and durations as well as effective diffusion times. Being aware of such uncertainties, it seems reasonable to apply the ADC measurements of STEAM-DWI as an alternative to the ADC values of EPI-DWI. If desired, a conversion factor of about 13% may be applied to STEAM-DWI ADC values to compensate for the systematic deviation to EPI-DWI ADC values under the chosen experimental conditions.

The CNR of STEAM-DWI in our study reached only 52% of that of EPI-DWI. This statistically significant difference between both sequences may partly be caused by a much lower T2 weighting, but in any case results in less conspicuous hyperintense regions in the isoDWI images in STEAM-DWI than in EPI-DWI. Khalil et al. came to a similar conclusion with a CNR of STEAM-DWI of 47% of that of EPI-DWI [7]. In practice, this requires the isoDW images of STEAM-DWI to be inspected more carefully for putative signal increases, as those pathological findings do not emerge as clearly as in EPI-DWI. A possible explanation arises from differences in the duration of the diffusion-weighted spin-echo interval. Whether longer TE values (i.e., a stronger T2 weighting) improves the situation for STEAM-DWI remains to be seen in a future study involving multiple acquisitions with different spin-echo times.

The lower visual resolution of STEAM-DWI compared to EPI-DWI is to be expected because the latter sequence benefits from an increased resolution of 0.6 mm due to interpolation by zero filling. It remains to be seen whether further optimization of the iterative image reconstruction or the adaptation of a suitable interpolation technique will be able to improve image sharpness for STEAM-DWI.

A limitation of the study is that the comparison of the STEAM-DWI was performed with a conventional EPI-DWI and not with variants more resistant to susceptibility, such as RESOLVE-DWI [10] or PROPELLER-DWI [11]. However, these sequences are not yet readily available on a regular basis in many institutions. In addition, the utility of the STEAM-DWI in the pediatric patient population described here is not diminished by the fact that there are possible alternatives to this sequence. Another limitation is the absence of a structured placement of ROI for the comparison of the ADC values of the STEAM-DWI and EPI-DWI. However, a structured elicitation in defined brain regions would result in clusters instead of widespread dispersion, which would be disadvantageous for the formation of a regression. The fact that the patients chosen to sample the ADC values were of different ages and of different pathologies contributes positively to the variance of the ADC values and thus favors the validity of the regression. Lastly, it should also be mentioned that STEAM-DWI benefits from a field strength of 3 T and the availability of high-performance gradients (80 mT/m). While a lower maximum gradient strength only prolongs the echo time of the spin-echo diffusion module, slower gradients impair the speed and SNR of the STEAM MRI part.

## Conclusion

Due to its lower CNR and slightly longer acquisition time, STEAM-DWI has not been able to replace EPI-DWI as the standard diffusion sequence in our institute. However, for the assessment of processes of the brainstem, posterior fossa and orbit, as well as when increased susceptibility artifacts (e.g., due to braces) are to be expected, it is routinely used due to its high reliability and lack of artifacts.

## Supporting information

S1 Fig Example of STEAM-DWI with a) b0 and b) isoDWI image as well as c) ADC map in a six-year-old girl with seizures, but without pathologic finding in brain imaging. (TIF)

S2 Fig Assessment of subjective resolution on the basis of the mesencephalic aqueduct in a 12-year-old girl with a pineal gland cyst. While the aqueduct (arrow) can be well distinguished in the isoDW image of the a) EPI-DWI, it cannot be reliably visualized in the b) STEAM-DWI.
(TIF)

S3 Fig Assessment of diagnostic confidence on the example of a 15-year-old girl with multiple brain contusions after traffic accident in the isoDW image of a) EPI-DWI and b) STEAM-DWI. In STEAM-DWI the actual diffusion defect remains (arrow) while the artificial high signals at the brain-bone junction in EPI-DWI (arrowhead) vanish.
(TIF)

S4 Fig Assessment of artifact size in patients with shunt valves in an 8-year-old boy with ventriculoperitoneal shunt after infratentorial pilocytic astrocytoma. The signal drop-out in the isoDW in the a) EPI-DWI (yellow border) is not seen in the b) STEAM-DWI.
(TIF)

S5 Fig Assessment of percent contrast-to-noise ratio in a 5-year-old girl with anaplastic ependynoma of the posterior fossa and postoperative cerebellar infarction. The regions of interest in both a) EPI-DWI and b) STEAM-DWI were placed in the diffusion-restricted areas and, in flipped form, contralaterally in healthy tissue.
(TIF)

S6 Fig Assessment of ADC values in an 8-year-old boy with adrenoleukodystrophy. On the ADC map of a) EPI-DWI, freehand regions-of-interest were placed in homogeneous areas and transferred to the ADC map of b) STEAM-DWI.
(TIF)

S7 Fig ADC values obtained by EPI-DWI and STEAM-DWI as Bland-Altman plot. As shown in Fig 6, a systematic deviation between the ADC values of both sequences can be observed.
(TIFF)
