# Supplementary figures and images for: STEAM-DWI as a robust alternative to EPI-DWI: Evaluation in pediatric brain MRI
Source: PLoS One. 2022 May 18;17(5):e0268523. doi: 10.1371/journal.pone.0268523 (PMC9116624; doi:10.1371/journal.pone.0268523)

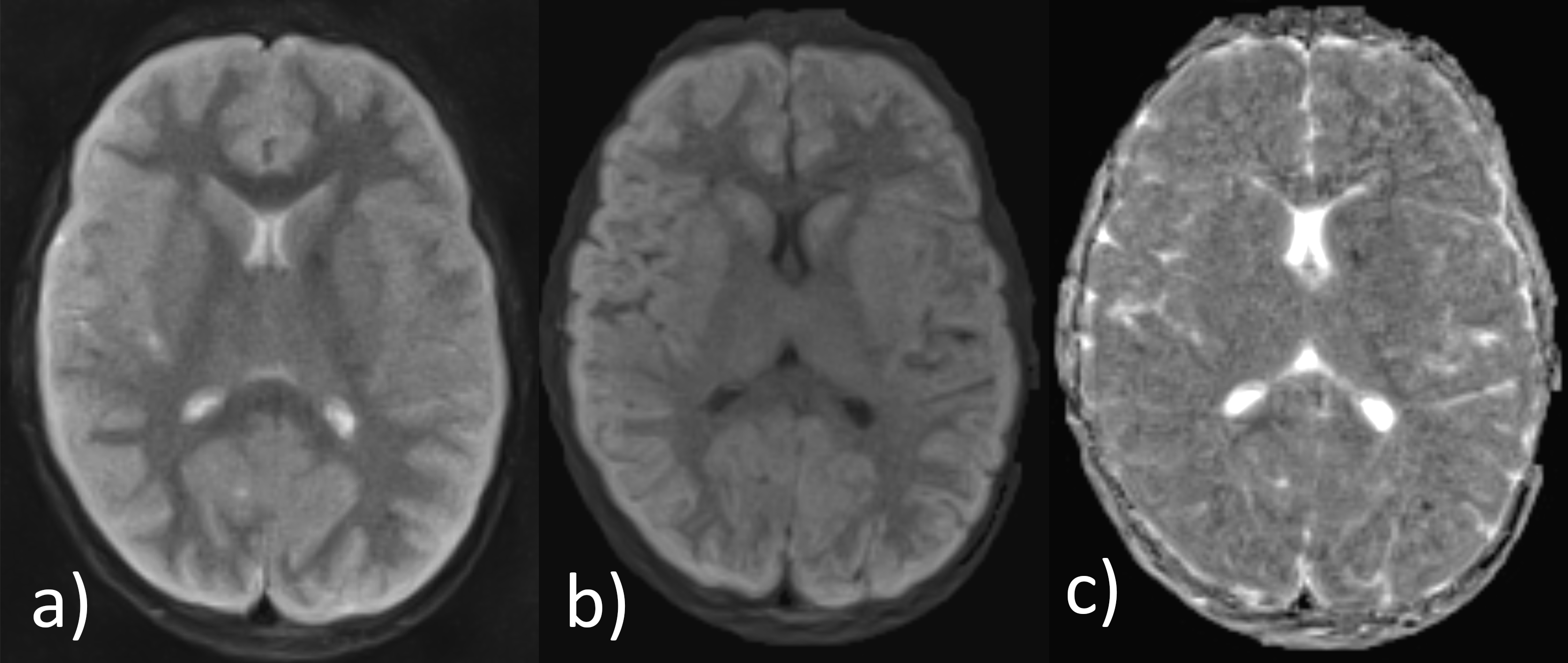

Supplement: S1 Fig — (TIF) [file pone.0268523.s001.tif]

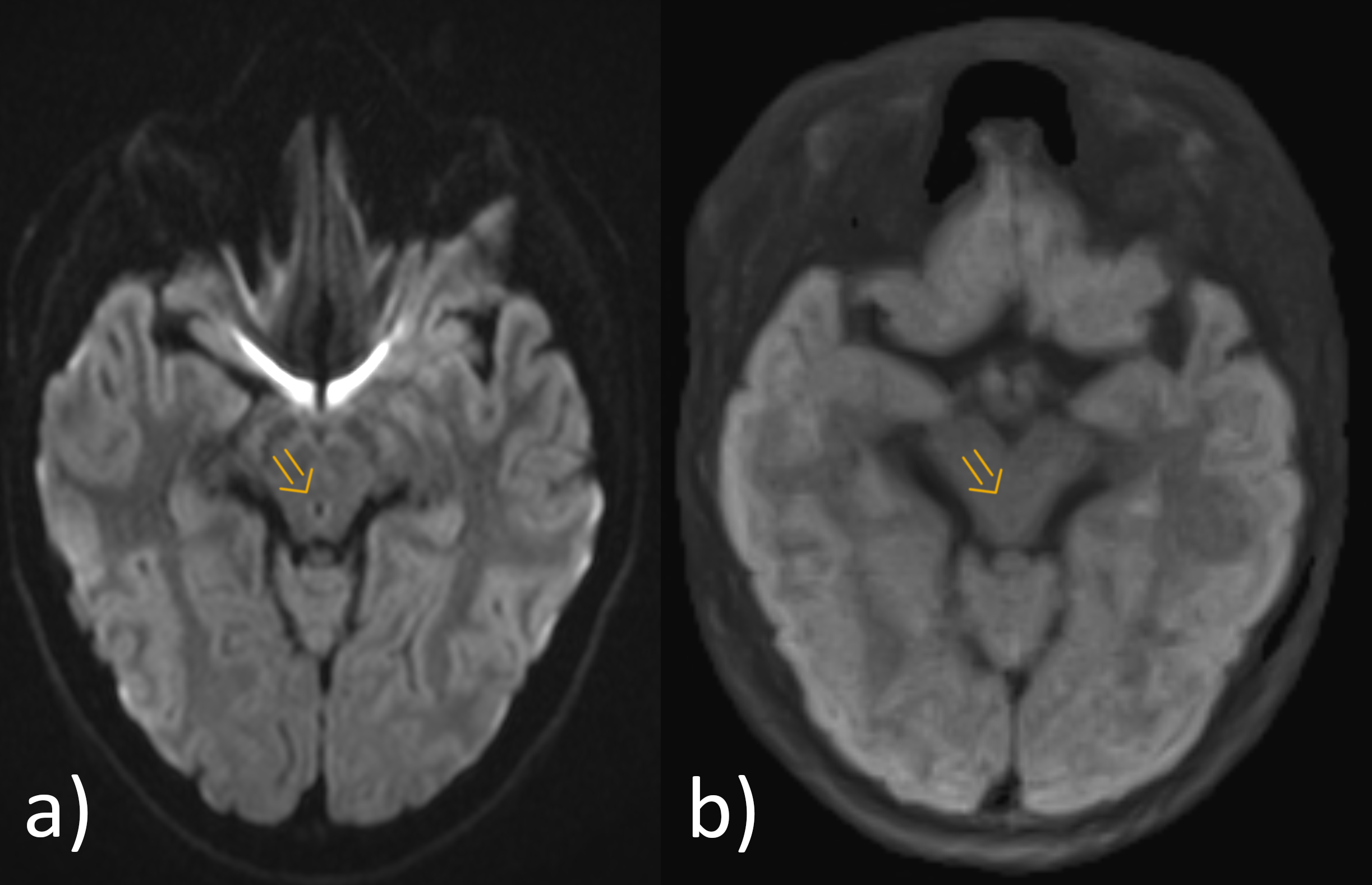

Supplement: S2 Fig — While the aqueduct (arrow) can be well distinguished in the isoDW image of the a) EPI-DWI, it cannot be reliably visualized in the b) STEAM-DWI. (TIF) [file pone.0268523.s002.tif]

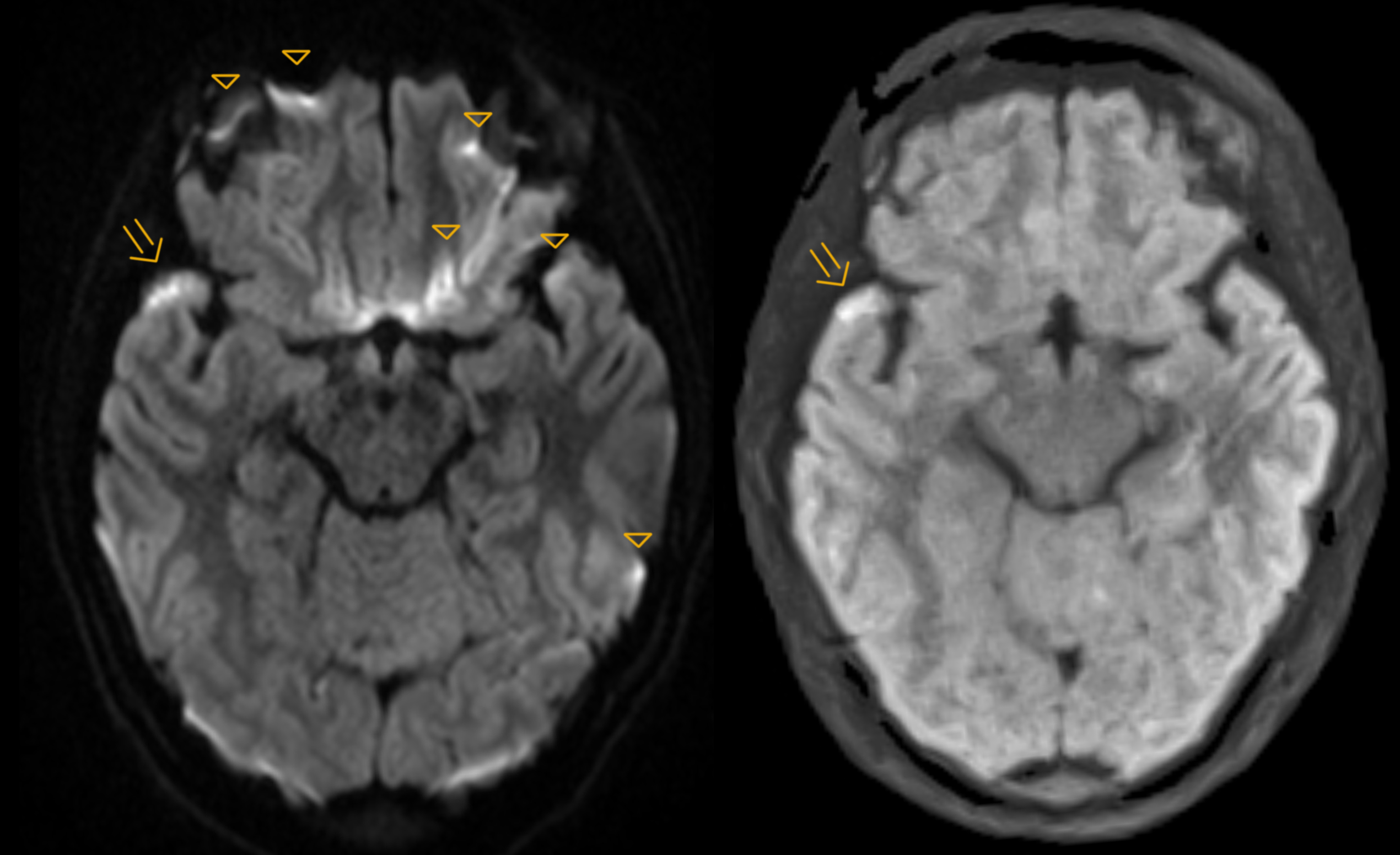

Supplement: S3 Fig — In STEAM-DWI the actual diffusion defect remains (arrow) while the artificial high signals at the brain-bone junction in EPI-DWI (arrowhead) vanish. (TIF) [file pone.0268523.s003.tif]

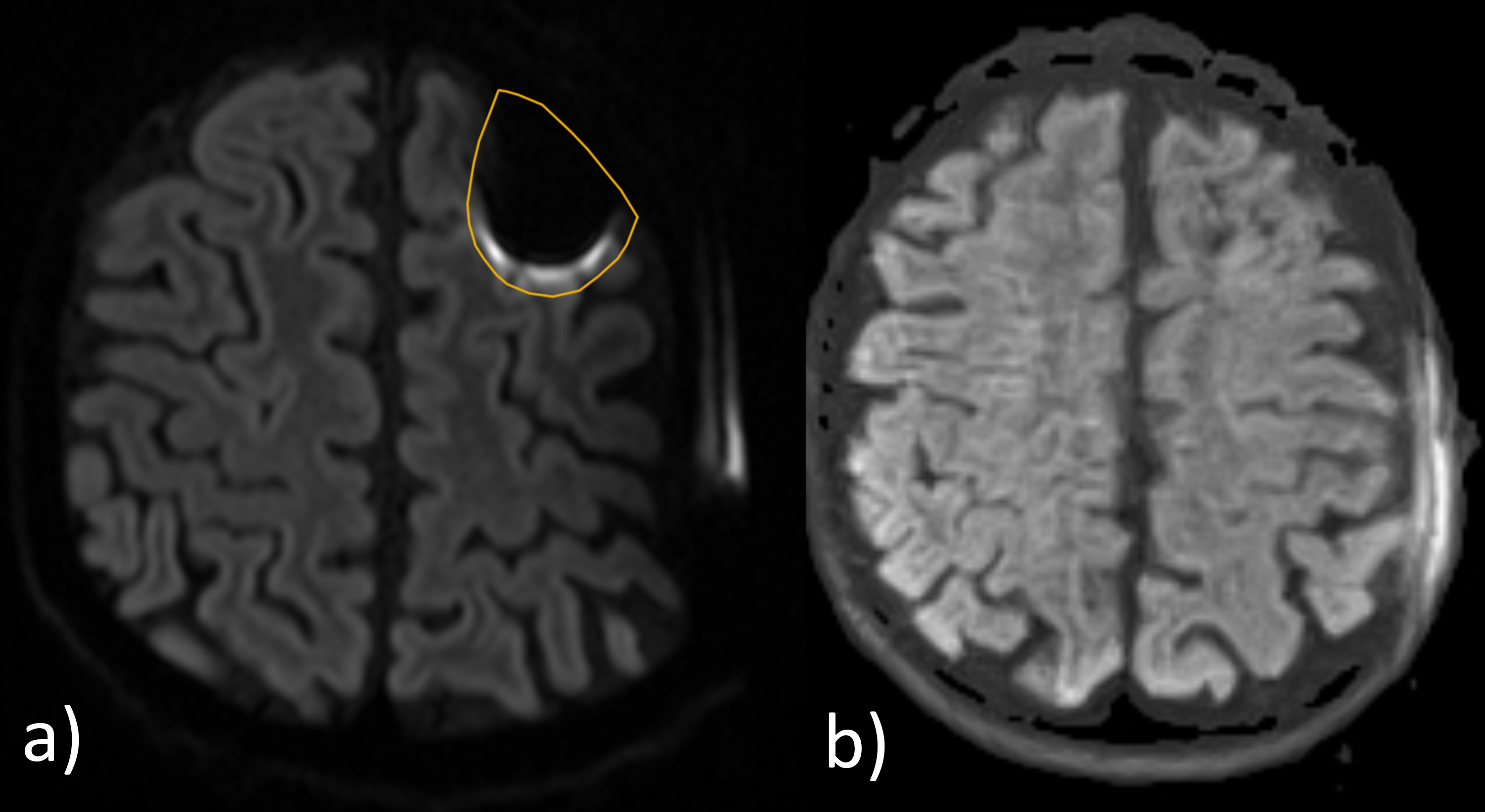

Supplement: S4 Fig — The signal drop-out in the isoDW in the a) EPI-DWI (yellow border) is not seen in the b) STEAM-DWI. (TIF) [file pone.0268523.s004.tif]

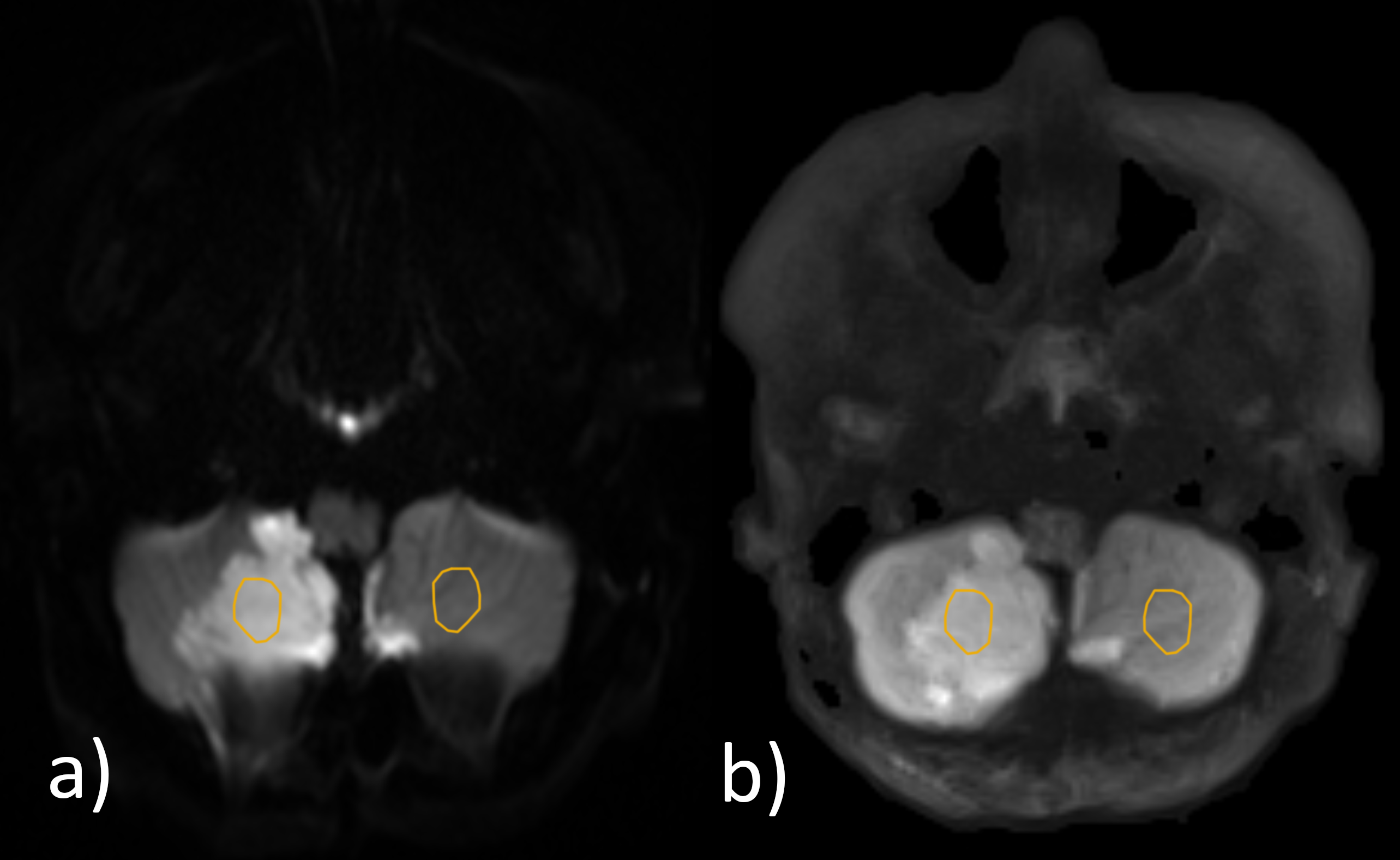

Supplement: S5 Fig — The regions of interest in both a) EPI-DWI and b) STEAM-DWI were placed in the diffusion-restricted areas and, in flipped form, contralaterally in healthy tissue. (TIF) [file pone.0268523.s005.tif]

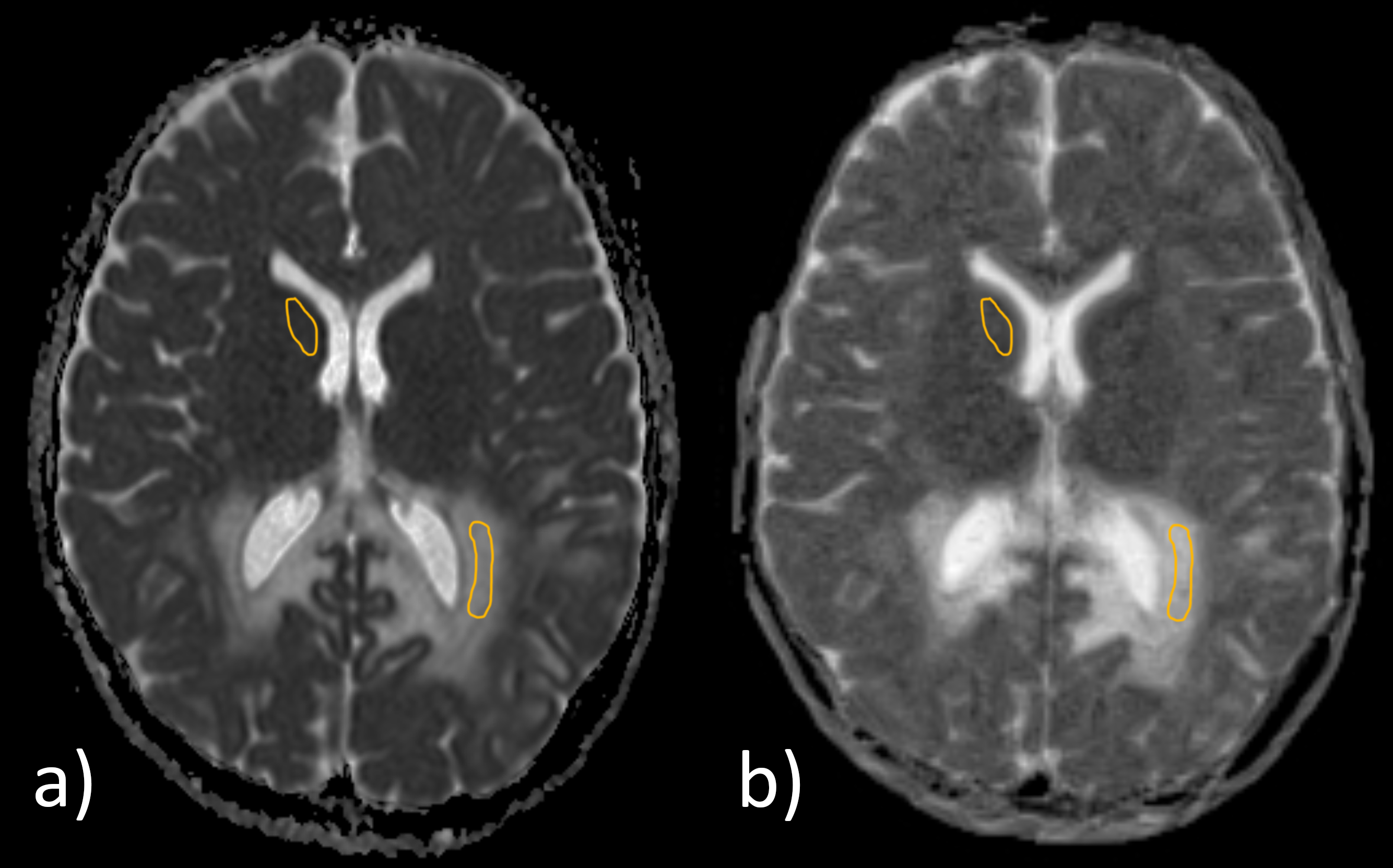

Supplement: S6 Fig — On the ADC map of a) EPI-DWI, freehand regions-of-interest were placed in homogeneous areas and transferred to the ADC map of b) STEAM-DWI. (TIF) [file pone.0268523.s006.tif]

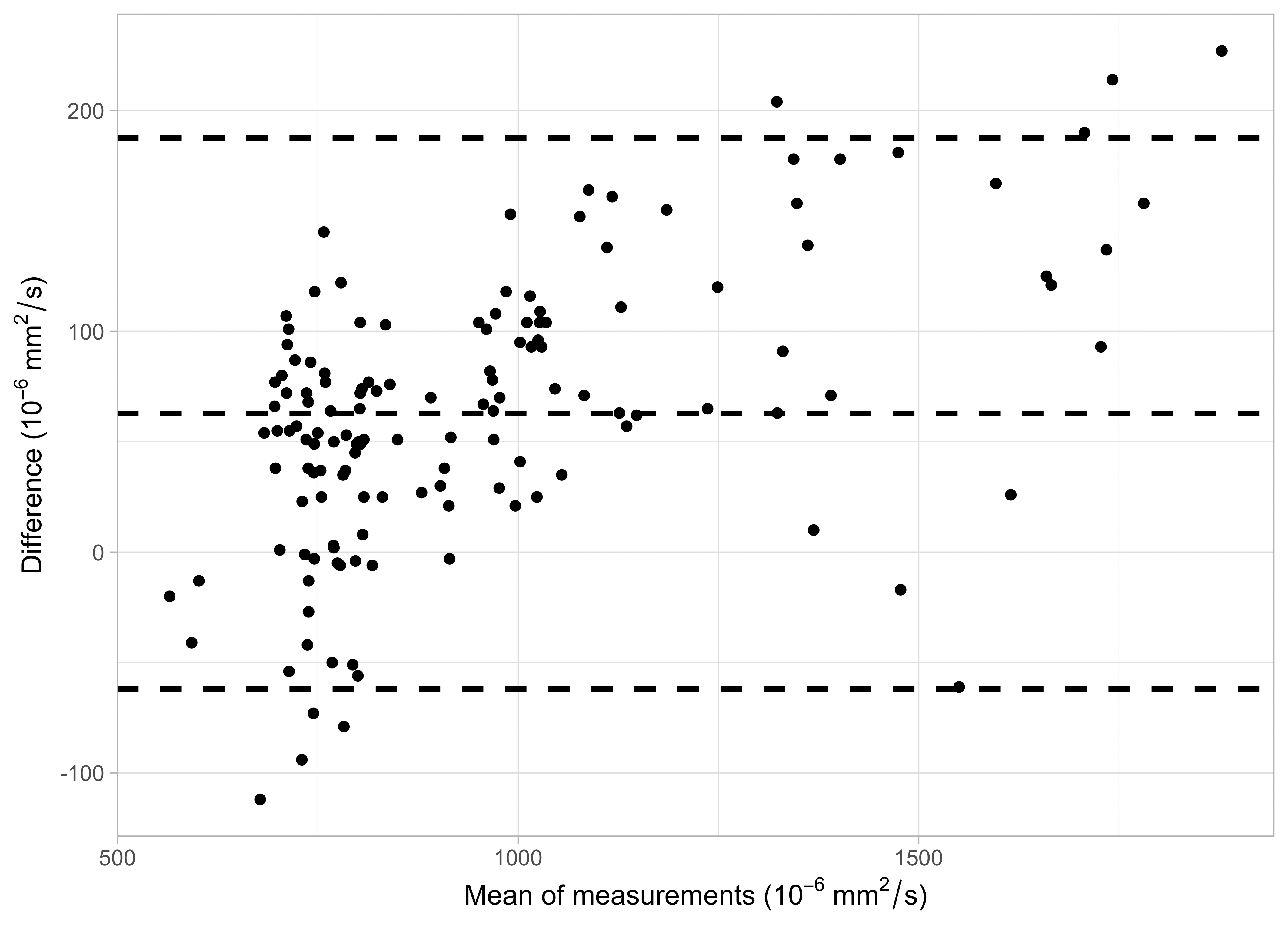

Supplement: S7 Fig — As shown in Fig 6, a systematic deviation between the ADC values of both sequences can be observed. (TIFF) [file pone.0268523.s007.tiff]
